# Analysis of the AUDIT factor structure in adolescents between 18 and 19 years

**DOI:** 10.11606/s1518-8787.2021055002777

**Published:** 2021-05-13

**Authors:** Patrícia Maria Abreu Machado, Cleber Lopes Campelo, João Victor Pimentel de Oliveira, Rosângela Fernandes Lucena Batista, Vanda Maria Ferreira Simões, Alcione Miranda dos Santos

**Affiliations:** I Universidade Federal do Maranhão Programa de Pós-Graduação em Ciências da Saúde São LuísMA Brasil Universidade Federal do Maranhão. Programa de Pós-Graduação em Ciências da Saúde. São Luís, MA. Brasil; II Universidade Federal do Maranhão Programa de Pós-Graduação em Saúde Coletiva São LuísMA Brasil Universidade Federal do Maranhão. Programa de Pós-Graduação em Saúde Coletiva. São Luís, MA, Brasil; III Universidade Federal do Maranhão Faculdade de Medicina São LuísMA Brasil Universidade Federal do Maranhão. Faculdade de Medicina. São Luís, MA, Brasil; IV Universidade Federal do Maranhão Departamento de Saúde Pública São LuísMA Brasil Universidade Federal do Maranhão. Departamento de Saúde Pública. São Luís, MA, Brasil

**Keywords:** Validation Study, Factor Analysis, Statistical, Underage Drinking, Alcoholic Beverages, Triage

## Abstract

**OBJECTIVE:**

To determine the factor structure of the instrument Alcohol Use Disorders Identification Test (AUDIT) in a representative sample of adolescents aged 18 to 19 years.

**METHODS:**

Cross-sectional study performed with adolescents born in São Luís (MA). The internal consistency of the instrument was determined by the Cronbach's alpha coefficient, and the validity of the construct was assessed by Confirmatory Factor Analysis (CFA). The Kaiser-Meyer-Olkin (KMO) was estimated to analyze the adequacy of the sample. The fit quality of the factor model was analyzed according to the indexes of the Chi-square adjustment test, comparative fit index (CFI), Tucker-Lewis index (TLI) and root mean square error of approximation (RMSEA).

**RESULTS:**

The sample of the study was composed of 1,002 adolescents aged from 18 to 19 years, being 56.8% girls, 68.5% with 18 years, 63.3% brown, 48.6% belonging to class C, 15.4% did not work or did not study, and 52.1% had divorced parents. The sample was suitable for confirmatory factor analysis (KMO = 0.79); Cronbach's alpha coefficient was 0.70, demonstrating satisfactory internal consistency with factor loads above 0.5, except for item 9, “was injured or someone else was injured due to drinking.” Confirmatory factor analysis revealed the validity of the three-factor model for the studied sample based on the indices of psychometric adjustments.

**CONCLUSION:**

The three-factor AUDIT factor structure was confirmed for the population of adolescents between 18 and 19 years old living in São Luís, ratifying the original conceptual domains proposed by the World Health Organization. AUDIT proved to be a reliable instrument to identify the consumption of alcohol.

## INTRODUCTION

Excessive alcohol consumption during adolescence can cause several health impairments in both biological and psychological, social and economic dimensions^1^. Early alcohol consumption results in impairments in school performance, adoption of risky behaviors, such as illicit drug use, smoking, early pregnancy, violence and traffic accidents^2^.

According to data from the *Pesquisa Nacional de Saúde do Escolar* (PeNSE – National School Health Survey)^3^ conducted in 2012, which evaluated 109,104 students of the 9th year of public and private elementary schools of the 26 capitals and the Federal District, 66.6% of the students had already experienced alcoholic beverage and, of these, 50.3% responded that they have consumed alcohol at least once in their life. This study also revealed that girls (51.7%) had a higher proportion of alcohol experimentation than boys (48.7%).

Faced with this reality, the World Health Organization (WHO) has encouraged the development and use of instruments to detect and measure the consumption of alcohol and other psychoactive substances^4^. Among the internationally recommended instruments, the following stand out: the Alcohol Use Disorders Identification Test (AUDIT), the Car, Relax, Alone, Forget, Family, Friends, Trouble (CRAFFT); the Cut-down, Annoyed by Criticism, Guilty and Eye-opener (CAGE), and the Alcohol, Smoking and Substance Involvement Screening Test (ASSIST), all translated and subjected to a validation process in many countries^4^^,^^5^.

Among the instruments recommended by WHO, AUDIT stands out, which has been used internationally by clinicians and researchers in several population studies^6^^,^^7^^,^^9^^,^^10^ to identify risk groups and track inappropriate alcohol use. In order to determine psychometric performance, validation studies^8^^–^^12^ with the adult population and clinical samples have highlighted, in general, satisfactory results regarding the internal consistency, sensitivity and specificity of the instrument.

The original structure of the AUDIT shown by the WHO proposes three factors with the following theoretical domains^13^: “consumption” (items 1 to 3); “symptoms of dependence” (items 4 to 6); and “problems or consequences related to alcohol use” (items 7 to 10). However, there are studies^7^^,^^14^^,^^17^ developed in a population of adolescents who reported finding results of factor analysis referring to a structure with two factors, the first being related to “frequency” (items 1 to 3) and the second to “problems or consequences related to alcohol consumption” (items 4 to 10), with evidenced good internal consistency (α > 0.80).

Regarding the three-factor AUDIT model, a study conducted with Mexican adolescents and young adults aged 14 to 30 years showed more satisfactory adjustment rates than the two-factor model^18^. This same model was also confirmed by a comparative study^15^ between samples of young Americans and Filipinos, which showed that the factor structures of the AUDIT may be different according to gender and age. Although this study showed similar factor structure for the two samples, it was observed that the factor loads related to the consumption factors of Filipinos were significantly lower than those of the USA.

According to Lopez et al.^14^, Tulião et al.^15^ and Santos et al.^16^, evidence on the structure of the AUDIT with two or three factors, when applied in adolescents, has demonstrated satisfactory psychometric properties, recognizing the potential of the instrument to detect excessive alcohol consumption.

In Brazil, several researchers have conducted AUDIT validation studies^11^^,^^12^^,^^19^^,^^20^^,^^21^, applying it in different populations that attested good reproducibility, internal consistency and factor structure for the investigated contexts. Among the studies already conducted with Brazilian adolescents, an investigation by Mattara et al.^22^ stands out, which revealed good internal consistency and validity of the instrument; however, factor structure was not tested.

Brazilian literature shows a small volume of studies that aimed to analyze the AUDIT factor structure, in addition to the absence of a consensus on the most appropriate structural model, especially when applied in the adolescent population^8^. Studies involving the construction or adaptation of measures are scarcer when the age group is the end of adolescence, a period of greater vulnerability when adolescents experience the transition to adulthood, which demands answers to subjective, social and economic requirements^2^.

The analysis of the factor structure of the AUDIT in a sample of adolescents represents an important aspect for providing reliable information on the patterns of alcohol use. Thus, the objective of this study was to determine the factor structure of this instrument applied in adolescents from a city in northeastern Brazil.

## METHODS

A cross-sectional study was conducted with adolescents, of both sexes, aged between 18 and 19 years, born between 1997 and 1998 in public and private maternity hospitals participating in the birth cohort of the municipality of São Luís in the state of Maranhão.

The first stage of the cohort carried out between March 1997 and February 1998 took place in ten public and private hospitals in the city and accounted for 2,541 births. In the second stage of the cohort between 2005 and 2006, children aged 7 to 9 years were monitored, composing a final sample of 673 children. The third stage was performed in 2016, characterized by monitoring adolescents between 18 and 19 years, evaluated in the three segments of the research, composing a sample of 684 individuals. In order to increase the power of the sample and prevent future losses, the cohort was opened and included 1,831 new volunteers, totaling 2,515 individuals. The detailed methodological aspects can be seen in Simões et al.^23^.

This study only included adolescents who participated in the third moment of the cohort and who answered “yes” to the screening question: “Have you ever taken alcoholic beverage such as beer, wine, cachaça, liquor, champagne or whiskey?” If the answer was “yes,” the adolescent was invited to answer the AUDIT, individually and without the presence of the applicator to avoid the influence of other people in their answers.

Adolescents who did not fill the AUDIT completely were excluded from the study, as well as variables of interest that referred to sociodemographic and economic characteristics such as: age, sex, skin color, occupation, marital status of parents and position in social class considering the *Critério de Classificação Econômica do Brasil* (CCEB)^24^. Thus, the final sample resulted in 1,002 adolescents.

AUDIT is an instrument consisting of ten items with structured answers, on an ordinal scale, on the harmful or risky consumption of alcohol in the last 12 months. The score is obtained by adding the options that the respondent points out, totaling up to 40 points. The first eight questions have five answer possibilities, with values ranging from zero to four, and the last two with only three answer possibilities, with values from zero to four^13^.

Given the interest in studying the AUDIT factor structure applied in individuals in the final phase of adolescence, descriptive statistical procedures were performed to characterize the sociodemographic profile of the sample. Then, the confirmatory factor analysis (CFA) of the instrument was performed in order to evaluate the structural consistency of the AUDIT with different factors: a single factor (items 1 to 10), with two factors, being Factor 1 called “Frequency” (items 1 to 3) and Factor 2 “Problems or consequences related to alcohol consumption” (items 4 to 10), and with three factors, being Factor 1 “Consumption” (items 1 to 3), Factor 2 “Symptoms of dependence” (items 4 to 6) and Factor 3 “Negative consequences related to alcohol” (items 7 to 10)^13^.

To evaluate the AUDIT factor structure and choose the most adjusted model, the following statistical indicators^25^ were used: root mean square error of approximation (RMSEA), comparative fit index (CFI) and Tucker-Lewis index (TLI). The models that showed CFI and TLI values greater than 0.5 and RMSEA lower than 0.05 were considered more suitable for the study sample. We also considered the ratio between the Chi-square of the model and its degrees of freedom (χ^2^*/gl*), with 2 and 3 being considered as reference values for an adequate fit, and the factor load greater than 0.5 for item selection^25^. The matrix of polychoric correlations, via weighted least squares mean and variance-adjusted (WLSMV) method^25^, was used in the CFA.

To evaluate the statistical difference between model adjustments, we used the Scaled Chi-Squared Difference Test^26^. To determine the internal consistency, we estimated the Cronbach's alpha coefficients^27^, as well as the Kaiser-Meyer-Olkin (KMO) measure^25^, to verify the adequacy of the sample. All analyses were performed in the R statistical program (version 3.2.4), with the help of the Lavaan package^28^ to perform the CFA.

The study was approved by the Research Ethics Committee of the Hospital Universitário of the Universidade Federal do Maranhão, protocol no. 1.302.489.

## RESULTS

Among the 1,002 adolescents (Table 1) included in the study, the majority were 18 years old (66.9%), and 569 (56.8%) were female. In relation to color/race, 634 (63.3%) declared themselves brown, 213 (21.2%) white and 155 (15.5%) black. Regarding the occupation of adolescents and the marital status of parents, 848 (84.6%) adolescents studied or worked and 522 (52.1%) had married parents or in a stable union. Regarding social class, 84 (9.4%) belonged to Class A, 436 (48.9%) to Class B, 358 (40.2%) to Class C and 13 (1.5%) to Class D/E.

**Table 1:** Sociodemographic characteristics of adolescents who responded to the AUDIT (n = 1,002).

Variable		Answered the AUDIT (n = 1,002)
		f (%)
Age		
	18 years	670 (66.9%)
	19 years	332 (33.1%)
Sex		
	Female	569 (56.8%)
	Male	433 (43.2%)
Social class		
	A	84 (9.4%)
	B	436 (48.9%)
	C	358 (40.2%)
	D/E	13 (1.5%)
Color/race		
	Brown	634 (63.3%)
	White	213 (21.2%)
	Black	155 (15.5%)
	Yellow/Asian	0 (0.0)
Marital status of parents		
	Divorced	480 (47.9%)
	Married or stable union	522 (52.1%)
Occupation (work or study)		
	Does not study or work	154 (15.4%)
	Studies or works	848 (84.6%)

We observed that 44.2% of adolescents reported consuming alcoholic beverages between two and four times a month, 32.5% reported consuming one or two doses of alcohol on a normal day and 37.5% reported consuming six or more doses on a single occasion at least once a month. In addition, 86% of the sample did not increase their alcohol consumption after starting drinking, 85% never failed to perform usual tasks because they drank, and 93.5% of the sample also never needed to consume alcohol to cure a hangover, as shown in Table 2.

**Table 2:** Frequency of responses to AUDIT items (n = 1,002).

		f	%
**1. How often do you consume drinks that contain alcohol?**			
	Never.	0	0.0
	Once a month or less	415	41.4
	2 to 4 times a month.	443	44.2
	2 to 3 times a week.	118	11.7
	4 or more times a week.	26	2.59
**2. When you drink, how many alcohol-containing drinks do you consume on a normal day?**			
	1 or 2 doses.	326	32.5
	3 or 4 doses.	135	13.4
	5 or 6 doses.	207	20.6
	7 to 9 doses.	70	7
	10 or more doses.	264	26.3
**3. How often do you consume six or more drinks on a single occasion?**			
	Never.	152	15.1
	Less than once a month.	280	27.9
	At least once a month.	376	37.5
	At least once a week.	172	17.7
	Daily or almost daily.	22	2.2
**4. In the last 12 months, how often did you realize that you couldn't stop drinking after you started?**			
	Never.	862	86
	Less than once a month.	47	4.6
	At least once a month.	60	6
	At least once a week.	26	2.5
	Daily or almost daily.	7	0.7
**5. In the last 12 months, how often have you failed to fulfill the tasks you are usually required to do because you had been drinking?**			
	Never.	850	85
	Less than once a month.	72	7.2
	At least once a month.	64	6.3
	At least once a week.	12	1.2
	Daily or almost daily.	4	0.4
**6. In the last 12 months, how often did you need to drink early in the morning to “cure” a hangover?**			
	Never.	937	93.5
	Less than once a month.	28	2.7
	At least once a month.	21	2.1
	At least once a week.	12	1.2
	Daily or almost daily.	4	0.4
**7. In the past 12 months, how often have you felt guilt or remorse for drinking?**			
	Never.	758	75.6
	Less than once a month.	104	10.3
	At least once a month.	112	11.1
	At least once a week.	19	1.9
	Daily or almost daily.	9	0.9
**8. In the last 12 months, how often did you not remember what happened the night before because of drinking?**			
	Never.	686	68.4
	Less than once a month.	152	15.1
	At least once a month.	124	12.3
	At least once a week.	31	3
	Daily or almost daily.	9	0.9
**9. Have you ever been hurt or has someone been hurt because you drank?**			
	No.	921	92
	Yes, but not in the last 12 months.	28	2.8
	Yes, it happened in the last 12 months.	53	5.3
**10. Has a family member, friend, doctor or health care professional ever expressed concern about your drinking or suggested that you stop drinking?**			
	No.	742	74
	Yes, but not in the last 12 months.	26	2.5
	Yes, it happened in the last 12 months.	234	23.3

The results also showed that 75.6% reported never feeling guilty or remorseful for drinking and that 68.4% remembered what they did after drinking alcohol. 92% of adolescents reported no problems with negative consequences for themselves or others due to alcohol use, or even having been called to attention due to alcohol consumption, as observed in 74% of adolescents.

Table 3 shows the distributions of the factor loads of the items for the models with one, two and three factors and the statistical indices used to evaluate the quality of the models analyzed in this study.

**Table 3:** Factor load of items for models adjusted with one-, two- and three-factor AUDIT (n = 1,002).

ITEMS	1 Factor	2 Factors		3 Factors		
	F1	F1	F2	F1	F2	F3
1	0.649	0.678		0.678		
2	0.520	0.573		0.575		
3	0.736	0.832		0.831		
4	0.663		0.722		0.763	
5	0.640		0.696		0.736	
6	0.517		0.563		0.592	
7	0.500		0.565			0.561
8	0.588		0.644			0.645
9	0.330		0.347			0.349
10	0.523		0.575			0.573

Adjustment indices	AUDIT factor structure		
	1 Factor	2 Factors	3 Factors
*x*^2^*/gl*	5.38	2.14	2.04
RMSEA	0.066	0.034	0.032
CFI	0.946	0.986	0.988
TLI	0.930	0.982	0.983

F1: consumption; F2: dependence; F3: negative consequences related to alcohol; *x*^2^/*gl*: ratio between the Chi-square of the model and the degree of freedom; RMSEA: root mean square error of approximation; CFI: comparative fit index; TLI: Tucker-Lewis index.

Factor loads were greater than 0.5 for most items in all models, except for item 9 — “Was injured or someone was injured due to drinking” —, which showed a factor load lower than 0.5 for the studied sample. Item 3 — “How often do you consume six or more drinks on a single occasion?” — showed the highest factor load in all models.

Considering the quality adjustment indicators of the models, it was observed that the unifactorial structure did not show satisfactory adjustment, demonstrating that this model was not suitable for the studied sample. Two-and three-factor structures showed more satisfactory adjustment rates.

When comparing the two- and three-factor models, they showed a statistically significant difference (*p* = 0.016), and the three-factor model was the most appropriate. The factorability of the correlation matrix was confirmed by measuring the KMO = 0.79, which revealed the adequacy of the sample. Considering the three-factor model, the Cronbach's alpha coefficient for the internal consistency of all items was 0.70 and, for each factor, it was: Factor 1 (Consumption) = 0.62; Factor 2 (Symptoms of dependence) = 0.52; Factor 3 (Negative consequences related to alcohol) = 0.41.

## DISCUSSION

This study evaluated the AUDIT factor structure in a representative sample of adolescents from São Luís, Maranhão. The results showed that the three-factor structure obtained a satisfactory adjustment for the studied sample, in line with the original conceptual domains^13^ proposed by the WHO and with other studies^10^^,^^11^^,^^15^^,^^18^ conducted with adolescents.

Cronbach's alpha coefficient for the total set of items was also consistent with what literature indicates as acceptable^27^^,^^30^, greater than or equal to 0.70; however, it is important to highlight that the second and third factors of the AUDIT had coefficients below this value.

For some research scenarios, a below-average Cronbach's Alpha coefficient may be considered acceptable provided that the results obtained with this instrument are interpreted considering other statistical measures^29^. The reliability value estimated by Cronbach's Alpha is not a specificity of the instrument; it is therefore an estimate of the reliability of the data being measured and that inform the accuracy of the instrument, and the values obtained are subject to the circumstances and the population where it was applied^30^. The reliability of an instrument is not a static measure, so lower Cronbach's alpha values do not impair the reliability of the instrument^29^.

Regarding Factor 1, the alpha coefficient for internal consistency was lower than acceptable. However, it was observed that the factor loads of the items that compose this factor were above 0.5, denoting that these items explain alcohol consumption. It is known that the higher the value of the factor load, the better the item represents the factor, thus indicating the existence of correlation between the items. These findings were similar to the results obtained in studies with adolescents from Ecuator^14^, the United States, Spain^17^ and Southern Mexico^18^, who obtained factor loads above 0.5 for this factor.

Cronbach's alpha coefficients for Factor 2 (Symptoms of alcohol dependence) and Factor 3 (Negative consequences that may occur from alcohol use) were below acceptable. This fact may be due to the homogeneity of the responses of the items that compose such factors. Thus, a high frequency of “never” responses, as occurred with this sample, may have influenced the extraction of factor loads resulting in values below the acceptable. A possible explanation for this is that these items may be describing situations and behaviors that are not part of the behavioral repertoire of these adolescents or that were poorly evaluated by the sample, which resulted in inconsistencies in the answers.

We observed that, in the items that compose Factor 2, the most frequent response was related to the non-occurrence of addiction symptoms. The lack of heterogeneity in the responses of Factor 3 items may have also reflected in the internal consistency of the instrument. However, these findings do not justify the elimination of the items that correspond to each factor, since the factor loads were above 0.5, except for item 9.

According to a study by Campo-Arias and Oviedo^29^, if in the evaluation of an item the majority of respondents tend to provide the same answer, there will be no great variability in this item and, therefore, reliability will be low; thus, to obtain measures with high reliability, there must be heterogeneity between the answers.

A study conducted by Tulião et al.^15^ with two samples from different countries revealed a three-factor structure for the AUDIT, while the frequency of item responses showed higher scores for the United States sample compared to the Philippines, indicating differences in the prevalence of alcohol consumption. In that study, the distribution of individual items indicated that the sample from the Philippines showed a greater tendency to lower scores, pointing out more homogeneity of item responses related to alcohol consumption, with Cronbach's Alpha coefficients below 0.70 for the three factors. In contrast, the United States sample showed a Cronbach's alpha value above 0.70 only for Factor 1, denoting a higher alcohol consumption for this population. This indicates that the factorial structure of the AUDIT may vary according to the population and culture where the instrument is applied.

The reliability findings of our study attest that the AUDIT may be a reliable tracking tool for application in adolescents, since studies with Cronbach's alpha above 0.70 were conducted with a population whose age group differs from the studied sample, which makes it difficult to compare it with other findings.

Regarding the factor load of item 9 of Factor 3 with a value below 0.5, this was also found by other studies conducted with adolescents from Ecuador^14^, the United States^7^^,^^15^, Philippines^15^ and Spain^17^, which suggests the withdrawal of this item, since they understand that younger consumers do not have many experiences resulting from negative consequences due to alcohol use. In this study, more than 90% of the responses regarding item 9 indicated the absence of situations that caused harm to themselves or third parties due to excessive alcohol consumption; the low variability of the responses may have influenced the factor load of this item below the expected^29^.

Several studies^7^^,^^16^^,^^17^^,^^18^ have emphasized the lack of uniformity regarding the AUDIT factor structures, and point out that this may result from the differences in sociocultural context, the characteristics of the sample and even the language, which may reflect in the conceptual framework underlying the AUDIT.

The tri-factor structure tested in this study allows staggering interventions so that they are planned and developed based on the complexity of problems related to alcohol consumption, making it a useful tool to implement prevention programs and enhance the offer of appropriate measures for the studied age group.

Considering alcohol as the gateway to other drug use, and teenage years as a time of increased psychosocial vulnerability^4^^,^^7^, we recommend that health care professionals perform interventions aimed at preventing and reducing alcohol consumption with feasible strategies that may be developed through the application of reliable instruments, such as the AUDIT, in order to track and detect patterns of alcohol consumption and, consequently, implement actions within a process that helps to reduce the problems and risks associated with it.

Although information on the use of alcohol alone is not sufficient to operate behavior changes, it becomes necessary for the construction of a perception about the risk of the effects associated with alcohol, being an important predictor for the planning of actions and decision-making.

Regarding the contributions of this study, we positively highlight the fact that it was conducted in a representative sample of adolescents aged 18 and 19 years whose frequency of consumption of alcoholic beverages was well above 40%, in addition to showing psychometric evidence regarding the tri-factor AUDIT model, which is in line with the model recommended by the WHO.

As limitations of this study, we point out that the sample analyzed covers a very specific age group, that is, the end of adolescence (18 to 19 years). Another limitation is that the data showed a Cronbach's alpha coefficient satisfactory only for the total AUDIT; however, these results do not substantially alter the reliability of the test. We recommend new studies with a population of adolescents more heterogeneous in relation to age, using the factor structure proposed by this study.

## CONCLUSION

The findings of this study show satisfactory psychometric properties regarding the three-factor AUDIT model in a sample of adolescents aged 18 and 19 years, being indicated as an appropriate instrument for screening in epidemiological studies aimed at investigating the pattern of alcohol use in this population.
