# Supplementary material for: Analysis of the AUDIT factor structure in adolescents between 18 and 19 years
Source: Rev Saude Publica. 2021 May 13;55:27. doi: 10.11606/s1518-8787.2021055002777 (PMC8139847; doi:10.11606/s1518-8787.2021055002777)
Supplement: Translation

# Translation: Análise da estrutura fatorial do Audit em adolescentes entre 18 e 19 anos

**Authors:** Patrícia Maria Abreu Machado, Cleber Lopes Campelo, João Victor Pimentel de Oliveira, Rosângela Fernandes Lucena Batista, Vanda Maria Ferreira Simões, Alcione Miranda dos Santos

**Affiliations:** I São LuísMA Brasil Universidade Federal do Maranhão. Programa de Pós-Graduação em Ciências da Saúde. São Luís, MA, Brasil; II São LuísMA Brasil Universidade Federal do Maranhão. Programa de Pós-Graduação em Saúde Coletiva. São Luís, MA, Brasil; III São LuísMA Brasil Universidade Federal do Maranhão. Faculdade de Medicina. São Luís, MA, Brasil; IV São LuísMA Brasil Universidade Federal do Maranhão. Departamento de Saúde Pública. São Luís, MA, Brasil

**Keywords:** Estudos de Validação, Análise Fatorial, Consumo de Álcool por Menores, Bebidas Alcoólicas, Triagem

## Abstract

**OBJETIVO:**

Determinar a estrutura fatorial do instrumento Alcohol Use Disorders Identification Test (Audit) em uma amostra representativa de adolescentes de 18 a 19 anos.

**MÉTODOS:**

Estudo transversal realizado com adolescentes nascidos em São Luís (MA). A consistência interna do instrumento foi determinada pelo coeficiente alfa de Cronbach e a validade do construto foi avaliada por meio da Análise Fatorial Confirmatória (AFC). O índice de Kaiser-Meyer-Olkin (KMO) foi calculado para analisar a adequação da amostra. A qualidade de ajuste do modelo fatorial foi analisada de acordo com os índices dos testes qui-quadrado de ajustamento, *comparative fit index* (CFI), *Tucker-Lewis index* (TLI) e *root mean square error of approximation* (RMSEA)*.*

**RESULTADOS:**

A amostra do estudo foi composta por 1.002 adolescentes entre 18 e 19 anos, sendo 56,8% meninas, 68,5% com 18 anos, 63,3% pardos ou mulatos, 48,6% pertencentes à classe C, 15,4% não trabalhavam e não estudavam e 52,1% tinham pais separados. A amostra foi adequada para a análise fatorial confirmatória (KMO = 0,79) e o coeficiente do alfa de Cronbach foi de 0,70, demostrando consistência interna satisfatória com cargas fatoriais acima de 0,5, com exceção do item 9 “Ficou ferido ou ficou alguém ferido por ter bebido”. A análise fatorial confirmatória revelou a validade do modelo de três fatores para a amostra em estudo com base nos índices de ajustes psicométricos.

**CONCLUSÃO:**

A estrutura fatorial do Audit com três fatores foi confirmada para a população de adolescentes entre 18 e 19 anos residentes em São Luís, ratificando os domínios conceituais originais propostos pela Organização Mundial da Saúde. O Audit apresentou-se como um instrumento confiável para a identificação do consumo de álcool.

## INTRODUÇÃO

O consumo excessivo de álcool durante a adolescência pode ocasionar diversos prejuízos à saúde tanto na dimensão biológica quanto psicológica, social e econômica^1^. A precocidade do consumo de álcool resulta em prejuízos no desempenho escolar, adoção de comportamentos de risco, como uso de drogas ilícitas, tabagismo, gravidez precoce, violência e acidentes de trânsito^2^.

Segundo dados da Pesquisa Nacional de Saúde do Escolar (PeNSE)^3^ realizada em 2012, que avaliou 109.104 alunos do 9° ano do ensino fundamental de escolas públicas e privadas das 26 capitais e do Distrito Federal, 66,6% dos escolares já haviam experimentado bebida alcoólica e destes 50,3% responderam que já consumiram álcool pelo menos alguma vez na vida. Esse estudo ainda revelou que meninas (51,7%) apresentavam uma proporção maior do que meninos (48,7%) quanto à experimentação de álcool.

Frente a essa realidade, a Organização Mundial da Saúde (OMS) tem incentivado o desenvolvimento e a utilização de instrumentos para detectar e mensurar o consumo de álcool e outras substâncias psicoativas^4^. Dentre os instrumentos internacionalmente recomendados, destacam-se o *Alcohol Use Disorders Identification Test* (Audit); o *Car, Relax, Alone, Forget, Family, Friends, Trouble* (CRAFFT); o *Cut down, Annoyed by Criticism, Guilty e Eye-opener* (CAGE); e o *Alcohol, Smoking and Substance Involvement Screening Test* (ASSIST), já traduzidos e submetidos a processos de validação em diversos países^4^^,^^5^.

Dentre os instrumentos recomendados pela OMS destaca-se o Audit, que tem sido utilizado internacionalmente por clínicos e investigadores em diversos estudos^6^^,^^7^^,^^9^^,^^10^ populacionais para identificação de grupos de risco e rastreamento do uso inadequado de álcool. Para determinação do desempenho psicométrico, estudos de validação^8^^–^^12^ com a população adulta e amostras clínicas têm destacado, no geral, resultados satisfatórios em relação a consistência interna, sensibilidade e especificidade do instrumento.

A estrutura original do Audit apresentada pela OMS propõe três fatores com os seguintes domínios teóricos^13^: “consumo” (itens 1 a 3); “sintomas de dependência” (itens 4 a 6); e “problemas ou consequências relacionadas ao uso de álcool” (itens 7 a 10). No entanto, existem estudos^7^^,^^14^^,^^17^ desenvolvidos em população de adolescentes que relataram ter encontrado resultados da análise fatorial remetendo a uma estrutura com dois fatores, sendo o primeiro relacionado a “frequência” (itens 1 a 3) e o segundo a “problemas ou consequências relacionadas ao consumo de álcool” (itens 4 a 10), sendo evidenciada boa consistência interna (α > 0,80).

Em relação ao modelo trifatorial do Audit, estudo realizado com adolescentes e adultos jovens mexicanos na faixa etária de 14 a 30 anos apresentou índices de ajuste mais satisfatórios do que o modelo de dois fatores^18^. Esse mesmo modelo também foi confirmado pelo estudo^15^ comparativo entre amostras de jovens norte-americanos e filipinos que evidenciou a possibilidade das estruturas fatoriais do Audit serem diferentes em função do gênero e da idade. Embora este estudo tenha apresentado estrutura fatorial semelhante para as duas amostras, observou-se que as cargas fatoriais referentes aos fatores de consumo dos filipinos foram significativamente mais baixas do que as dos EUA.

De acordo com Lopez *et al*.^14^, Tulião *et al*.^15^ e Santos *et al*.^16^, evidências sobre a estrutura do Audit com dois ou três fatores, quando aplicado em adolescentes, têm demonstrado propriedades psicométricas satisfatórias reconhecendo a potencialidade do instrumento para a detecção do consumo excessivo de álcool.

No Brasil, vários pesquisadores têm realizado estudos de validação^11^^,^^12^^,^^19^^,^^20^^,^^21^ do Audit, aplicando em diferentes populações que atestaram boa reprodutibilidade, consistência interna e estrutura fatorial para os contextos investigados. Dentre os estudos já realizados com adolescentes brasileiros, destaca-se o de Mattara et al.^22^ que revelou boa consistência interna e validade do instrumento, contudo não foi testada a estrutura fatorial.

A literatura brasileira mostra um volume pequeno de estudos que se propuseram a analisar a estrutura fatorial do Audit, além da ausência de um consenso quanto ao modelo estrutural mais adequado, especialmente quando aplicado em população de adolescentes^8^. Os estudos que envolvem a construção ou adaptação de medidas são mais escassos quando a faixa etária é o final da adolescência, período de maior vulnerabilidade, em que os adolescentes vivenciam a transição para a fase adulta, que demanda respostas para exigências de ordem subjetiva, social e econômica^2^.

A análise da estrutura fatorial do Audit em uma amostra de adolescentes representa um aspecto importante por proporcionar o fornecimento de informações confiáveis sobre os padrões de uso de álcool. Desta forma, o objetivo deste estudo foi determinar a estrutura fatorial desse instrumento aplicado em adolescentes de uma cidade do nordeste brasileiro.

## MÉTODOS

Estudo transversal realizado com adolescentes, de ambos os sexos, com idades entre 18 e 19 anos, nascidos entre 1997 e 1998 em maternidades públicas e privadas participantes da coorte de nascimento do município de São Luís no estado do Maranhão.

A primeira etapa da coorte realizada entre março de 1997 e fevereiro de 1998 ocorreu em dez hospitais da rede pública e privada da cidade e contabilizou 2.541 nascimentos. Na segunda etapa da coorte entre 2005 a 2006, foram acompanhadas crianças na faixa etária de 7 a 9 anos compondo a amostra final de 673 crianças. A terceira etapa foi realizada em 2016, caracterizada por acompanhar adolescentes entre 18 e 19 anos, avaliados nos três segmentos da pesquisa, compondo uma amostra de 684 indivíduos. Com o intuito de aumentar o poder da amostra e prevenir perdas futuras, a coorte foi aberta e incluídos 1.831 novos voluntários, totalizando 2.515 indivíduos. O detalhamento dos aspectos metodológicos pode ser visto em Simões *et al*.^23^

Neste estudo foram incluídos apenas os adolescentes que participaram do terceiro momento da coorte e que responderam “sim” a pergunta de triagem: “Você já tomou bebida alcoólica como cerveja, vinho, cachaça, licor, champanha ou uísque?” Caso a resposta fosse “sim”, o adolescente era convidado a responder o Audit, de forma individual e sem a presença do aplicador para evitar a influência de outras pessoas em suas respostas.

Foram excluídos do estudo os adolescentes que não preencheram o Audit completamente, bem como as variáveis de interesse que se referiam às características sociodemográficas e econômicas como: idade, sexo, cor da pele, ocupação, situação conjugal dos pais e posição na classe social considerando o Critério de Classificação Econômica do Brasil (CCEB)^24^. Desta forma, a amostra final resultou em 1.002 adolescentes.

O Audit é um instrumento constituído por dez itens com respostas estruturadas, em escala ordinal, sobre o consumo de álcool nocivo ou de risco nos últimos 12 meses. A pontuação é obtida somando as opções que o respondente assinala, totalizando até 40 pontos. As oito primeiras questões apresentam cinco possibilidades de resposta, com valores que variam de zero a quatro, e as duas últimas, somente três possibilidades de respostas, com valores de zero a quatro^13^.

Dado o interesse em estudar a estrutura fatorial do Audit aplicado em indivíduos na fase final da adolescência, foram realizados procedimentos de estatística descritiva para caracterização do perfil sociodemográfico da amostra. Em seguida, foi realizada a análise fatorial confirmatória (AFC) do instrumento com o objetivo de avaliar a consistência estrutural do Audit com diferentes fatores: com um único fator (itens 1 a 10), com dois fatores sendo o Fator 1 denominado “Frequência” (itens 1 a 3) e o Fator 2 “Problemas ou consequências relacionadas ao consumo de álcool” (itens 4 a 10) e com três fatores sendo o Fator 1 “Consumo” (itens 1 a 3), Fator 2 “Sintomas de dependência” (itens 4 a 6) e Fator 3 “Consequências negativas relacionadas ao álcool” (itens 7 a 10)^13^.

Para avaliar a estrutura fatorial do Audit e escolher o modelo mais ajustado, foram utilizados os seguintes indicadores estatísticos^25^: *root mean square error of approximation* (RMSEA), *comparative fit index* (CFI) *e Tucker-Lewis index* (TLI).** Os modelos que apresentaram valores de CFI e TLI maiores do que 0,5 e RMSEA inferior a 0,05 foram considerados mais adequados para a amostra em estudo. Também foram consideradas a razão entre o qui-quadrado do modelo e seus graus de liberdade (χ^2^*/gl*) sendo considerados 2 e 3 como valores de referência para um ajuste adequado e a carga fatorial superior a 0,5 para seleção dos itens^25^. A matriz de correlações policóricas, via método *weighted least squares mean and variance-adjusted* (WLSMV)^25^, foi utilizada na AFC.

Para avaliar a diferença estatística entre os ajustes dos modelos, foi realizado o Scaled Chi-Squared Difference Test^26^. Para determinar a consistência interna, foram realizados os cálculos dos coeficientes de alpha de Cronbach^27^ bem como a medida de Kaiser-Meyer-Olkin (KMO)^25^, para verificar a adequação da amostra. Todas as análises foram realizadas no programa estatístico R (versão 3.2.4), com o auxílio do pacote Lavaan^28^ para executar a AFC.

O estudo foi aprovado pelo Comitê de Ética em Pesquisa do Hospital Universitário da Universidade Federal do Maranhão protocolo n. 1.302.489.

## RESULTADOS

Observa-se que entre os 1.002 adolescentes (Tabela 1) incluídos no estudo, a maioria tinha 18 anos (66,9%), sendo que 569 (56,8%) eram do sexo feminino. Em relação a cor/raça, 634 (63,3%) se autodeclaram pardos ou mulatos, 213 (21,2%) brancos e 155 (15,5%) como negros. Quanto à ocupação dos adolescentes e à situação conjugal dos pais, 848 (84,6%) adolescentes estudavam ou trabalhavam e 522 (52,1%) tinham pais casados ou em união estável. Em relação à classe social, 84 (9,4%) pertenciam à classe A, 436 (48,9%) à classe B, 358 (40,2%) à classe C e 13 (1,5%) à classe D/E.

**Tabela 1:** Características sociodemográficas dos adolescentes que responderam o Audit (n = 1.002).

Variável		Responderam o Audit (n = 1.002)
		f (%)
Idade		
	18 anos	670 (66,9%)
	19 anos	332 (33,1%)
Sexo		
	Feminino	569 (56,8%)
	Masculino	433 (43,2%)
Classe social		
	A	84 (9,4%)
	B	436 (48,9%)
	C	358 (40,2%)
	D/E	13 (1,5%)
Cor/raça		
	Parda/mulata	634 (63,3%)
	Branca	213 (21,2%)
	Negra	155 (15,5%)
	Amarelo/Oriental	0 (0,0)
Situação conjugal dos pais		
	Separados	480 (47,9%)
	Casados ou união estável	522 (52,1%)
Ocupação (trabalho ou estudo)		
	Não estuda nem trabalha	154 (15,4%)
	Estuda ou trabalha	848 (84,6%)

Observou-se que 44,2% dos adolescentes relataram consumir bebidas alcoólicas entre duas e quatro vezes por mês, 32,5% referiram consumir uma ou duas doses de álcool em um dia normal e 37,5% referiram consumir seis doses ou mais numa única ocasião pelo menos uma vez por mês. Além disso, 86% da amostra responderam negativamente ao aumento do consumo de álcool após terem iniciado o consumo, 85% nunca deixaram de cumprir tarefas habituais por terem bebido e 93,5% da amostra também nunca precisou consumir álcool para curar uma ressaca, como apresentado na Tabela 2.

**Tabela 2:** Frequência de respostas aos itens do Audit (n = 1.002).

		f	%
**1. Com que frequência consome bebidas que contêm álcool?**			
	Nunca.	0	0.0
	1 vez por mês ou menos.	415	41.4
	2 a 4 vezes por mês.	443	44.2
	2 a 3 vezes por semana.	118	11.7
	4 ou mais vezes por semana.	26	2.59
**2. Quando bebe, quantas bebidas contendo álcool consome num dia normal?**			
	1 ou 2 doses.	326	32.5
	3 ou 4 doses.	135	13.4
	5 ou 6 doses.	207	20.6
	7 a 9 doses.	70	7
	10 ou mais doses.	264	26.3
**3. Com que frequência consome seis bebidas ou mais numa única ocasião?**			
	Nunca.	152	15.1
	Menos de 1 vez por mês.	280	27.9
	Pelo menos 1 vez por mês.	376	37.5
	Pelo menos 1 vez por semana.	172	17.7
	Diariamente ou quase diariamente.	22	2.2
**4. Nos últimos 12 meses, com que frequência você percebeu que não conseguia parar de beber depois de começar?**			
	Nunca.	862	86
	Menos de 1 vez por mês.	47	4.6
	Pelo menos 1 vez por mês.	60	6
	Pelo menos 1 vez por semana.	26	2.5
	Diariamente ou quase diariamente.	7	0.7
**5. Nos últimos 12 meses, com que frequência você não conseguiu cumprir as tarefas que habitualmente lhe exigem por ter bebido?**			
	Nunca.	850	85
	Menos de 1 vez por mês.	72	7.2
	Pelo menos 1 vez por mês.	64	6.3
	Pelo menos 1 vez por semana.	12	1.2
	Diariamente ou quase diariamente.	4	0.4
**6. Nos últimos 12 meses, com que frequência precisou beber logo de manhã para “curar” uma ressaca?**			
	Nunca.	937	93.5
	Menos de 1 vez por mês.	28	2.7
	Pelo menos 1 vez por mês.	21	2.1
	Pelo menos 1 vez por semana.	12	1.2
	Diariamente ou quase diariamente.	4	0.4
**7. Nos últimos 12 meses, com que frequência sentiu culpa ou remorso por ter bebido?**			
	Nunca.	758	75.6
	Menos de 1 vez por mês.	104	10.3
	Pelo menos 1 vez por mês.	112	11.1
	Pelo menos 1 vez por semana.	19	1.9
	Diariamente ou quase diariamente.	9	0.9
**8. Nos últimos 12 meses, com que frequência não se lembrou do que aconteceu na noite anterior por causa de ter bebido?**			
	Nunca.	686	68.4
	Menos de 1 vez por mês.	152	15.1
	Pelo menos 1 vez por mês.	124	12.3
	Pelo menos 1 vez por semana.	31	3
	Diariamente ou quase diariamente.	9	0.9
**9. Alguma vez já ficou ferido ou alguém foi ferido por você ter bebido?**			
	Não.	921	92
	Sim, mas não nos últimos 12 meses.	28	2.8
	Sim, aconteceu nos últimos 12 meses.	53	5.3
**10. Alguma vez um familiar, amigo, médico ou profissional de saúde já manifestou preocupação pelo seu consumo de álcool ou sugeriu que deixasse de beber?**			
	Não.	742	74
	Sim, mas não nos últimos 12 meses.	26	2.5
	Sim, aconteceu nos últimos 12 meses.	234	23.3

Os resultados ainda demonstraram que 75,6% relataram que nunca tiveram sentimento de culpa ou remorso por terem bebido e que 68,4% lembravam do que fizeram após o consumo de álcool. 92% dos adolescentes relataram ausência de problemas com consequências negativas para si ou para terceiros devido ao uso de álcool, ou mesmo terem sido chamados à atenção em função do consumo de álcool, como observado em 74% dos adolescentes.

Na Tabela 3 são apresentadas as distribuições das cargas fatoriais dos itens para os modelos com um, dois e três fatores e os índices estatísticos utilizados para avaliar a qualidade dos modelos analisados neste estudo.

**Tabela 3:** Carga fatorial dos itens para os modelos ajustados com um, dois e três fatores do Audit (n = 1.002).

ITENS	1 Fator	2 Fatores		3 Fatores		
	F1	F1	F2	F1	F2	F3
1	0,649	0,678		0,678		
2	0,520	0,573		0,575		
3	0,736	0,832		0,831		
4	0,663		0,722		0,763	
5	0,640		0,696		0,736	
6	0,517		0,563		0,592	
7	0,500		0,565			0,561
8	0,588		0,644			0,645
9	0,330		0,347			0,349
10	0,523		0,575			0,573

Índices de ajustes	Estrutura fatorial do Audit		
	1 Fator	2 Fatores	3 Fatores
*x*^2^/*gl*	5,38	2,14	2,04
RMSEA	0,066	0,034	0,032
CFI	0,946	0,986	0,988
TLI	0,930	0,982	0,983

F1: consumo; F2: dependência; F3: consequências negativas relacionadas ao álcool; *x*^2^/*gl*: razão entre o qui-quadrado do modelo e o grau de liberdade; RMSEA: *root mean square error of approximation*; CFI: *comparative fit index*; TLI: Tucker-Lewis index.

As cargas fatoriais foram superiores a 0,5 para a maioria dos itens em todos os modelos, exceto para o item 9 – “Ficou ferido ou ficou alguém ferido por ter bebido” –, que apresentou carga fatorial inferior a 0,5 para a amostra em estudo. Já o item 3 – “Com que frequência consome seis bebidas ou mais numa única ocasião” – apresentou a carga fatorial mais alta em todos os modelos.

Considerando os indicadores de ajuste de qualidade dos modelos, observou-se que a estrutura unifatorial não apresentou ajuste satisfatório, demonstrando que este modelo não foi adequado para a amostra em estudo. Já as estruturas com dois e três fatores apresentaram índices de ajuste mais satisfatórios.

Quando comparados os modelos com dois e três fatores, houve diferença estatisticamente significante (*p* = 0,016), mostrando que o modelo de três fatores foi o mais adequado. A fatorabilidade da matriz de correlação foi confirmada por meio da medida do KMO = 0,79 que revelou adequação da amostra. Considerando o modelo de três fatores, o coeficiente do alfa de Cronbach para a consistência interna de todos os itens foi de 0,70 e para cada fator foi: Fator 1 (Consumo) = 0,62; Fator 2 (Sintomas de dependência) = 0,52; Fator 3 (Consequências negativas relacionadas ao álcool) = 0,41.

## DISCUSSÃO

O presente estudo avaliou a estrutura fatorial do Audit em uma amostra representativa de adolescentes de São Luís, Maranhão. Os resultados mostraram que a estrutura com três fatores obteve ajuste satisfatório para a amostra em estudo, em consonância com os domínios conceituais originais^13^ propostos pela OMS e com outros estudos^10^^,^^11^^,^^15^^,^^18^ realizados com adolescentes.

O coeficiente alpha de Cronbach para o conjunto total de itens também foi condizente com o que a literatura aponta como aceitável^27^^,^^30^, maior ou igual a 0,70, contudo, é importante destacar que o segundo e o terceiro fator do Audit apresentaram coeficientes abaixo desse valor.

Para alguns cenários de investigação, um coeficiente alfa de Cronbach abaixo da média pode ser considerado aceitável desde que os resultados obtidos com esse instrumento sejam interpretados considerando outras medidas estatísticas^29^. O valor da confiabilidade estimada pelo alfa de Cronbach não é uma especificidade do instrumento, é, portanto, uma estimativa de confiabilidade dos dados que estão sendo mensurados e que informam a precisão do instrumento, estando os valores obtidos sujeitos as circunstâncias e a população onde foi aplicado^30^. A confiabilidade de um instrumento não é uma medida estática, portanto, valores mais baixos do alfa de Cronbach não inviabilizam a confiabilidade do instrumento^29^.

Em relação ao Fator 1, o coeficiente alfa referente à consistência interna foi inferior ao aceitável. Contudo, observou-se que as cargas fatoriais dos itens que compõem esse fator foram acima de 0,5, denotando que esses itens explicam o consumo de álcool. Sabe-se que quanto maior o valor da carga fatorial, melhor o item representa o fator, indicando assim a existência de correlação entre os itens. Esses achados foram semelhantes aos resultados obtidos em estudos com adolescentes do Equador^14^, dos Estados Unidos, da Espanha^17^ e do sul do México^18^, que obtiveram cargas fatorais acima de 0,5 para esse fator.

Os coeficientes do alfa de Cronbach para o Fator 2 (Sintomas de dependência do consumo de álcool) e Fator 3 (Consequências negativas que podem ocorrer pelo uso de álcool) foram abaixo do aceitável. Este fato pode ser devido à homogeneidade das respostas dos itens que compõem tais fatores. Assim, uma alta frequência de repostas de “nunca”, como ocorreu com essa amostra, pode ter influenciado na extração das cargas fatoriais resultando em valores abaixo do aceitável. Uma possível explicação para isso é que esses itens podem estar descrevendo situações e comportamentos que não fazem parte do repertório comportamental desses adolescentes ou, ainda, que foram mal avaliados pela amostra, o que resultou em inconsistências nas repostas.

Observou-se que, nos itens que compõem o Fator 2, a resposta mais frequente estava relacionada à não ocorrência de sintomas de dependência. A falta de heterogeneidade nas respostas dos itens do Fator 3 pode ter refletido também na consistência interna do instrumento. Entretanto, esses achados não justificam a eliminação dos itens que correspondem a cada fator, visto que as cargas fatoriais se apresentaram acima de 0,5, com exceção do item 9.

Segundo estudo de Campo-Arias e Oviedo^29^, se na avaliação de um item a maioria dos entrevistados tendem a fornecer a mesma resposta, não haverá grande variabilidade neste item e, por conseguinte, a confiabilidade será baixa, dessa forma, para obter medidas com alta confiabilidade, é preciso existir heterogeneidade entre as respostas.

Estudo realizado por Tulião *et al*.^15^ com duas amostras provenientes de países distintos revelou uma estrutura trifatorial para o Audit e a frequência de respostas dos itens apresentaram pontuações mais altas para a amostra dos Estados Unidos em comparação com a das Filipinas, indicando diferenças na prevalência do consumo de álcool. Nesse estudo, a distribuição dos itens individuais indicou que a amostra das Filipinas apresentou maior tendência para pontuações mais baixas, indicando mais homogeneidade das respostas para os itens relacionados ao consumo de álcool, revelando coeficientes de alfa de Cronbach abaixo de 0,70 para os três fatores. Em contrapartida, a amostra dos Estados Unidos apresentou o valor de alfa de Cronbach acima de 0,70 apenas para o Fator 1, denotando um maior consumo de álcool para essa população. Isto indica que a estrutura fatorial do Audit pode variar de acordo com a população e a cultura onde o instrumento for aplicado.

Os achados de confiabilidade do presente estudo atestam que o Audit pode ser um instrumento de rastreamento confiável para aplicação em adolescentes, visto que estudos que apresentam o alfa de Cronbach acima de 0,70 foram realizados com população cuja faixa etária difere da amostra em estudo, o que dificulta sua comparação com outros achados.

Com relação à carga fatorial do item 9 do Fator 3 com valor abaixo de 0,5, também já foi apresentado por outros estudos realizados com adolescentes do Equador^14^, dos Estados Unidos^7^^,^^15^, das Filipinas^15^ e da Espanha^17^, que sugeriram a retirada desse item por entender que consumidores mais jovens não têm muitas experiências decorrentes de consequências negativas devido ao uso de álcool. Neste estudo, mais de 90% das respostas referentes ao item 9 indicaram ausência de situações que tenham causado prejuízos para si ou terceiros em função do consumo excessivo de álcool, essa baixa variabilidade das respostas pode ter influenciado na carga fatorial desse item abaixo do esperado^29^.

Vários estudos^7^^,^^16^^,^^17^^,^^18^ tem enfatizado a ausência de uniformidade quanto às estruturas fatoriais do Audit e apontam que isso pode ser resultante das diferenças de contexto sociocultural, das características da amostra e até mesmo do idioma, que pode refletir no arcabouço conceitual subjacente ao Audit.

A estrutura trifatorial testada neste estudo possibilita o escalonamento das intervenções para que estas sejam planejadas e desenvolvidas a partir da complexidade dos problemas referentes ao consumo de álcool, tornando-a uma ferramenta útil para a implantação de programas de prevenção e potencializando a oferta de medidas adequadas para a faixa etária em estudo.

Considerando o álcool como a porta de entrada para o uso de outras drogas e a adolescência como um período de grande vulnerabilidade psicossocial^4^^,^^7^, recomenda-se aos profissionais de saúde a realização de intervenções que visem à prevenção e redução do consumo de álcool por meio de estratégias factíveis que possam ser desenvolvidas por meio da aplicação de instrumentos confiáveis, como o Audit, a fim de realizar o rastreamento e detectar os padrões de consumo de álcool e, consequentemente, a implementação de ações dentro de um processo que auxilie a redução dos problemas e riscos associados.

Embora as informações sobre o uso de álcool isoladamente não sejam suficientes para operar mudanças de comportamento, se tornam necessárias para a construção de uma percepção sobre o risco dos efeitos associados ao álcool, sendo um importante preditor para o planejamento de ações e tomada de decisões.

Em relação às contribuições deste estudo, destaca-se positivamente o fato de ter sido realizado em uma amostra representativa de adolescentes com 18 e 19 anos cuja frequência de consumo de bebidas alcoólicas foi muito acima de 40%, além de apresentar evidências psicométricas quanto ao modelo trifatorial do Audit, que está em consonância com o modelo recomendado pela OMS.

Como limitações deste estudo, aponta-se que a amostra analisada abrange uma faixa etária muito específica, isto é, o final de adolescência (18 a 19 anos). Outra limitação é que os dados evidenciaram um coeficiente alfa de Cronbach satisfatório apenas para o Audit total, entretanto, esses resultados não alteram substancialmente a confiabilidade do Audit. Recomenda-se novos estudos com uma população de adolescentes mais heterogênea em relação à idade utilizando a estrutura fatorial proposta por este estudo.

## CONCLUSÃO

Os achados do presente estudo evidenciam propriedades psicométricas satisfatórias quanto ao modelo de três fatores do Audit em uma amostra de adolescentes com 18 e 19 anos, sendo indicado como instrumento adequado para o rastreamento em estudos epidemiológicos, visando investigar o padrão de uso de álcool nessa população.
